# Polymer Nanoformulation of Sorafenib and All-Trans Retinoic Acid for Synergistic Inhibition of Thyroid Cancer

**DOI:** 10.3389/fphar.2019.01676

**Published:** 2020-02-03

**Authors:** Shijie Li, Shujun Dong, Weiguo Xu, Yang Jiang, Zhongmin Li

**Affiliations:** ^1^ Department of Thyroid Surgery, China-Japan Union Hospital of Jilin University, Changchun, China; ^2^ VIP Integrated Department, School and Hospital of Stomatology, Jilin University, Changchun, China; ^3^ Key Laboratory of Polymer Ecomaterials, Changchun Institute of Applied Chemistry, Chinese Academy of Sciences, Changchun, China; ^4^ Department of Gastrointestinal Colorectal and Anal Surgery, China-Japan Union Hospital of Jilin University, Changchun, China

**Keywords:** sorafenib, all-trans retinoic acid, drug delivery system, polymer micelles, thyroid cancer

## Abstract

Part of differentiated thyroid cancer will relapse or develop into dedifferentiated thyroid cancer after standard therapy, such as surgery or radionuclide therapy. Sorafenib (SOR) is recommended for the treatment of advanced or radioiodine-refractory thyroid cancer. The monotherapy using SOR is often hampered by its modest efficacy, serve systemic toxicity, and high occurrence of drug resistance. In order to enhance the antitumor effect of SOR and reduce its side effects, SOR and all-trans retinoic acid (ATRA), a differentiation-promoting drug, were loaded into poly(ethylene glycol)–poly(lactide-*co*-glycolide) (PEG–PLGA) polymer micelles in this study. The drug-loaded micelles, PM/(SOR+ATRA), exhibited relatively slow drug release and effective cell uptake. Compared with other treatment groups, the PM/(SOR+ATRA) treatment group showed the most significant antitumor effect and minimal systemic toxicity toward the FTC-133 thyroid cancer-bearing BALB/c nude mouse model. Immunofluorescence analysis confirmed that PM/(SOR+ATRA) could significantly promote apoptosis and re-differentiation of tumor cells. All the results demonstrated that polymer micelles loaded with SOR and ATRA could treat thyroid cancer more effectively and safely.

## Introduction

As the most common endocrine malignancy, thyroid cancer has become more and more prevalent in recent years (James et al., 2018). Differentiated thyroid cancer (DTC), which originates from follicular epithelial cells, accounts for more than 95% of thyroid cancer and usually has a good prognosis (Cabanillas et al., 2016). Surgery and postoperative thyrotropin suppression therapy or radionuclide therapy (I^131^) are currently the standard treatment for DTC (Haugen et al., 2016). However, approximately 30% of these patients will relapse after the standard treatment (Kim et al., 2018b). The recurrent DTC tends to lose the ability to absorb iodine, thereby losing the option of radioactive iodine therapy. Multi-targeted kinase inhibitors such as sorafenib (SOR) are the first-line treatment of radioiodine-refractory DTC (RAIR-DTC) or advanced thyroid cancer, and they can significantly improve the progression-free survival of patients (Kim et al., 2018a). SOR exhibits an excellent antitumor effect on various thyroid cancer cell lines, including dedifferentiated subtypes, leading to tumor cell apoptosis and cell cycle arrest (Broecker-Preuss et al., 2015). Nevertheless, drug resistance and serious adverse events, including hand-foot skin reactions, diarrhea, and hypertension, limit the clinical application of SOR (Wei et al., 2019). Studies have shown that patients with DTC have a higher incidence of adverse events to SOR compared to patients with renal and hepatocellular cancer, and about half of the patients need to reduce the dose to control drug toxicity in phase 2 and 3 trials (Jean et al., 2016). Therefore, how to improve the antitumor effect and reduce the adverse events of SOR is an urgent problem to be solved.

In addition, some DTCs will dedifferentiate into more aggressive types after standard treatment. Studies have shown that most of the anaplastic thyroid cancer or poorly differentiated thyroid cancer is derived from the dedifferentiation of DTC (Ma et al., 2018). These dedifferentiated thyroid cancers have become a serious clinical problem due to their lack of response to conventional treatments such as radiotherapy or chemotherapy. Differentiation therapy, which enables dedifferentiated tumor cells to continue to differentiate and mature by using differentiation inducers, is a promising strategy to treat dedifferentiated thyroid cancer. All-trans retinoic acid (ATRA), an intermediate metabolite of vitamin A, has the ability to inhibit cell proliferation and metastasis and promote cell differentiation and apoptosis (Cui et al., 2016). ATRA has been widely used in the differentiation therapy of various diseases, such as acute promyelocytic leukemia (Burnett et al., 2015), breast cancer (Coyle et al., 2018), and advanced thyroid cancer (Cristiano et al., 2017). Studies have proved that ATRA could promote the re-differentiation of thyroid cancer cells, which was characterized by high expression of sodium iodide symporter (NIS) and increased cell uptake of ^131^I (Arisi et al., 2014).

Synergistic drug combinations are promising cancer treatment strategies, which can improve the therapeutic effect by integrating the functions of multiple drugs (Lehar et al., 2009; Stathias et al., 2018). However, there are still some problems in the clinical application, including poor specificity of drug distribution and serious systemic side effects (Boutros et al., 2016). Nanotechnology has provided a promising method for the targeted delivery of multiple drugs to tumor tissues, which can effectively solve the problems encountered in combination therapy with free drugs (Zhang et al., 2016a; Yang et al., 2017). Nanocarriers are characterized by good biocompatibility, high drug loading efficiency, prolonged circulation time *in vivo*, increased aggregation in tumor tissues, surface modification to actively target specific tissues and cells, stimuli-sensitive behavior for controlled drug release, and simultaneous delivery of different drugs for combination therapy (Feng et al., 2017; Xu et al., 2017; Guo et al., 2018; Zhang et al., 2018a; Feng et al., 2019; Wang et al., 2019). In recent years, a variety of multifunctional nanomaterials have been developed for co-delivery of multiple drugs, such as polymer nanoparticles (Zhao et al., 2016), micelles (Wang et al., 2018; Yang et al., 2019), liposomes (Yao et al., 2015), inorganic nanoparticles (Zhang et al., 2016b), and nanogels (Liu et al., 2015; Zhang et al., 2018b). These delivery systems can deliver drugs to tumor tissues more effectively and safely, thus improving the therapeutic effect.

In this study, we speculated that the re-differentiation effect induced by ATRA could enhance the antitumor efficiency of SOR, and the nanocarriers could further enhance the therapeutic effect. First, SOR and ATRA-loaded poly(ethylene glycol)–poly(lactide-*co*-glycolide) (PEG–PLGA) polymer micelles were fabricated and characterized. Then their antitumor efficiency toward FTC-133 thyroid cancer-bearing BALB/c nude mouse model was investigated. In addition, the systemic side effects were also assessed. The results demonstrated that ATRA significantly enhanced the antitumor efficiency of SOR against FTC-133 tumor cells *in vitro* and *in vivo*, and PEG–PLGA co-loaded with SOR and ATRA showed the most obvious tumor inhibition effect. Therefore, co-delivery of SOR and ARTA with nanocarriers is an effective treatment for thyroid cancer.

## Materials and Methods

### Materials

PEG [number-average molecular weight (*M*_n_) = 2,000 Da] and PLGA [L-lactide (LA):glycolide (GA) = 80:20; viscosity-average molecular weight (*M*_η_) = 60,000 Da] were obtained from Changchun SinoBiomaterials Co., Ltd. (Changchun, P. R. China). SOR (99%) and ATRA (98.5%) were obtained from Zhejiang Hisun Pharmaceutical Co., Ltd. (Taizhou, P. R. China). Stannous 2-ethylhexanoate [Sn(Oct)_2_, 95%] was provided by Sigma-Aldrich, Inc. (St. Louis, MO, USA). 4′,6-Diamidino-2-phenylindole (DAPI) and 3-(4,5-dimethylthiazol-2-y1)-2,5-diphenyltetrazolium bromide (MTT) were provided by Sigma-Aldrich (Shanghai, P. R., China). The primary and secondary antibodies of caspase-3, phosphorylated extracellular regulated protein kinases 2 (p-ERK2), thyroglobulin (Tg), and NIS were obtained from Abcam (Cambridge, MA, USA). Clear 6-well and 96-well cell culture plates were provided by Corning Costar Co. (Cambridge, MA, USA).

### Preparation of Drug-Loaded Polymer Micelles

PEG–PLGA copolymer was prepared according to our previous procedure (Li et al., 2013). SOR and ATRA were encapsulated into PEG–PLGA micelles through a nanoprecipitation method. Typically, PEG–PLGA copolymer (12.5 mg) and SOR (2.5 mg) were dissolved by 100.0 μL of dimethyl sulfoxide (DMSO), respectively. Then these two kinds of solutions were mixtured. After that, 0.90 mL of Milli-Q water was added into the mixed solution. The mixture was then stirred evenly at room temperature for 2 h and subsequently dialyzed with deionized water for 12 h. SOR-loaded PEG–PLGA polymer micelles (PM/SOR) were obtained after the product was lyophilized. For the synthesis of ATRA-loaded PEG–PLGA polymer micelles (PM/ATRA), 31.25 mg of PEG–PLGA copolymer and 6.25 mg of ATRA were dissolved in 100.0 μL of DMSO, respectively. And then, the synthesis process is the same as PM/SOR. SOR and ATRA-loaded PEG–PLGA polymer micelles [PM/(SOR+ATRA)] were prepared in the same way.

### Characterizations of Drug-Loaded Polymer Micelles

The sizes of PM/SOR, PM/ATRA, and PM/(SOR+ATRA) were analyzed by dynamic laser scattering (DLS) using a Wyatt QELS equipment (Wyatt Technology Corp., Santa Barbara, CA, USA). The morphology of drug-loaded PEG–PLGA micelles was observed by a JEOL JEW-1011 transmission electron microscope (TEM; Tokyo, Japan).

### *In Vitro* Drug Release

The drug release behavior of drug-loaded PEG–PLGA micelles (*i.e*., PM/SOR, PM/ATRA, and PM/(SOR+ATRA) was studied through a dialysis approach. In brief, 1.0 mg of drug-loaded PLGA micelles were dissolved in 10.0 mL of phosphate-buffered saline (PBS) at pH 7.4, and then they were placed in dialysis bags [molecular weight cut-off (MWCO) = 3,500 Da], respectively. The dialysis bags were put into 100.0 mL of corresponding PBS at 37°C and kept vibrating at 80 rpm. At predetermined time intervals, 2.0 mL of external release medium was extracted for detection, and then the same amount of PBS was added. The amount of released SOR and ATRA was calculated by measuring the UV-vis absorbance at 269.0 and 343.5 nm, respectively.

### *In Vitro* Cell Uptake

The *in vitro* cell uptake of drugs was evaluated in FTC-133 human thyroid cancer cells. In brief, the FTC-133 cells at a density of 2.0 × 10^5^ cells mL^–1^ were inoculated on a six-well plate and cultured at 37°C for 24 h. After that, each well was washed twice using PBS and incubated in FBS-absent Dulbecco’s Modified Eagle Medium (DMEM) with different concentrations of SOR, ATRA, SOR+ATRA, PM/SOR, PM/ATRA, and PM/(SOR+ATRA). In the control group, cells were incubated with PBS. After 2 h of incubation, the medium was taken away, and each well was washed three times using PBS. Subsequently, 1 mL of lysate was added into each well and cultured at room temperature for 20 min. Finally, the cells were suspended and centrifuged at 3,000 rpm for 5 min. 500 μL of supernatant was collected and determined by measuring the UV-vis absorbance at 269.0 and 343.5 nm, respectively.

### *In Vitro* Cytotoxicity Test

MTT assay was used to test the cytotoxicity of SOR and ATRA on FTC-133 cells and HepG2 cells. The concentration of SOR was 0.0015–100.0 μmol L^–1^ and the concentration ATRA was 0.0031–200.0 μmol L^–1^. In brief, the FTC-133 cells at a density of 4.0 × 10^3^ cells mL^–1^ were inoculated on a 96-well plate in 180.0 μL of DMEM and cultured at 37°C for 24 h. After that, 20.0 μL of various concentrations of SOR or ATRA solutions were placed into each well and incubated for 72 h. And then, 20.0 μL of MTT (5.0 mg mL^–1^) was added to each well. After 4 h of incubation, the medium was taken away, followed by the addition of 150.0 μL of DMSO. After 5 min of vibration, the absorbance of the medium was measured at 490 nm by a Bio-Rad 680 microplate reader. Furthermore, the antitumor activity of the combination of SOR and ATRA with SOR at 18.0 μmol L^–1^ and ATRA ranging from 2.8 to 70.0 μmol L^–1^ was also evaluated on FTC-133 cells according to the above protocol. The cytotoxicity of SOR and ATRA to HepG2 cells was assessed using the same procedure. The cell viability was calculated using Equation (1).

(1) $$Cell\;viability\;(\%)=\frac{A_{sample}}{A_{control}}\times 100\%$$

*A*_sample_ and *A*_control_ represented the absorbances of sample and control wells, respectively.

### *In Vivo* Antitumor Efficacy Assessment

BALB/c nude mice (male, 8–12 weeks) were provided by the Animal Center of Jilin University and maintained at Changchun Institute of Applied Chemistry, Chinese Academy of Sciences. The animal studies were approved, and all the experiments were carried out under the supervision of the Animal Care and Use Committee at Jilin University.

FTC-133 cells (1 × 10^6^ cells mL^–1^) were inoculated in the right axillary of male BALB/c nude mice to establish the tumor-bearing mouse model. Once the tumor volume increased to approximately 100 mm^3^, the tumor-bearing mice were randomized into seven groups (*n* = 5 per group). These seven groups were treated with natural saline (as a control), SOR, ATRA, SOR+ATRA, PM/SOR, PM/ATRA, or PM/(SOR+ATRA) at equivalent SOR dose of 10.0 mg (kg BW) ^−1^ and ATRA dose of 25.0 mg (kg BW) ^−1^ by tail vein injection every 4 days for three times. Tumor size and body weight of each mouse were measured and recorded every day. Tumor volume was calculated using Equation (2).

(2) $$V(mm^{3})=\frac{L\times S^{2}}{2}$$

*L* and *S* (mm) represented the largest and smallest axial lengths of tumors, respectively.

### Histology Analysis and Immunofluorescence Assays

The mice were sacrificed using conventional cervical dislocation after 12 days of treatment. Tumors and major organs including heart, liver, spleen, lung, and kidney were resected and fixed with 10% neutral buffered formalin overnight and stained with hematoxylin and eosin staining (H&E) for histological observation and immunofluorescence analyses (*i.e*., caspase-3, Tg, NIS, and p-ERK2). The histological and immunofluorescence changes were assessed using a microscope (Nikon Eclipse *Ti*, Optical Apparatus Co., Ardmore, PA, USA). The tumor necrosis area and the fluorescent images were further quantitatively analyzed by Image J software (National Institutes of Health, USA). The fluorescence intensity of the control group was set as “1”.

### Statistical Analysis

Data were presented as mean ± standard deviation (SD). Statistical analyses were carried out by Student’s t-test with the statistical software SPSS (Version 21.0, SPSS Inc., Chicago, IL, USA). *P < 0.05 was considered statistically significant, and **P < 0.01 and ***P < 0.001 were considered highly statistically significant.

## Results and Discussion

### Synthesis and Characterization of Drug-Loaded PEG–PLGA Micelles

**Scheme 1:**
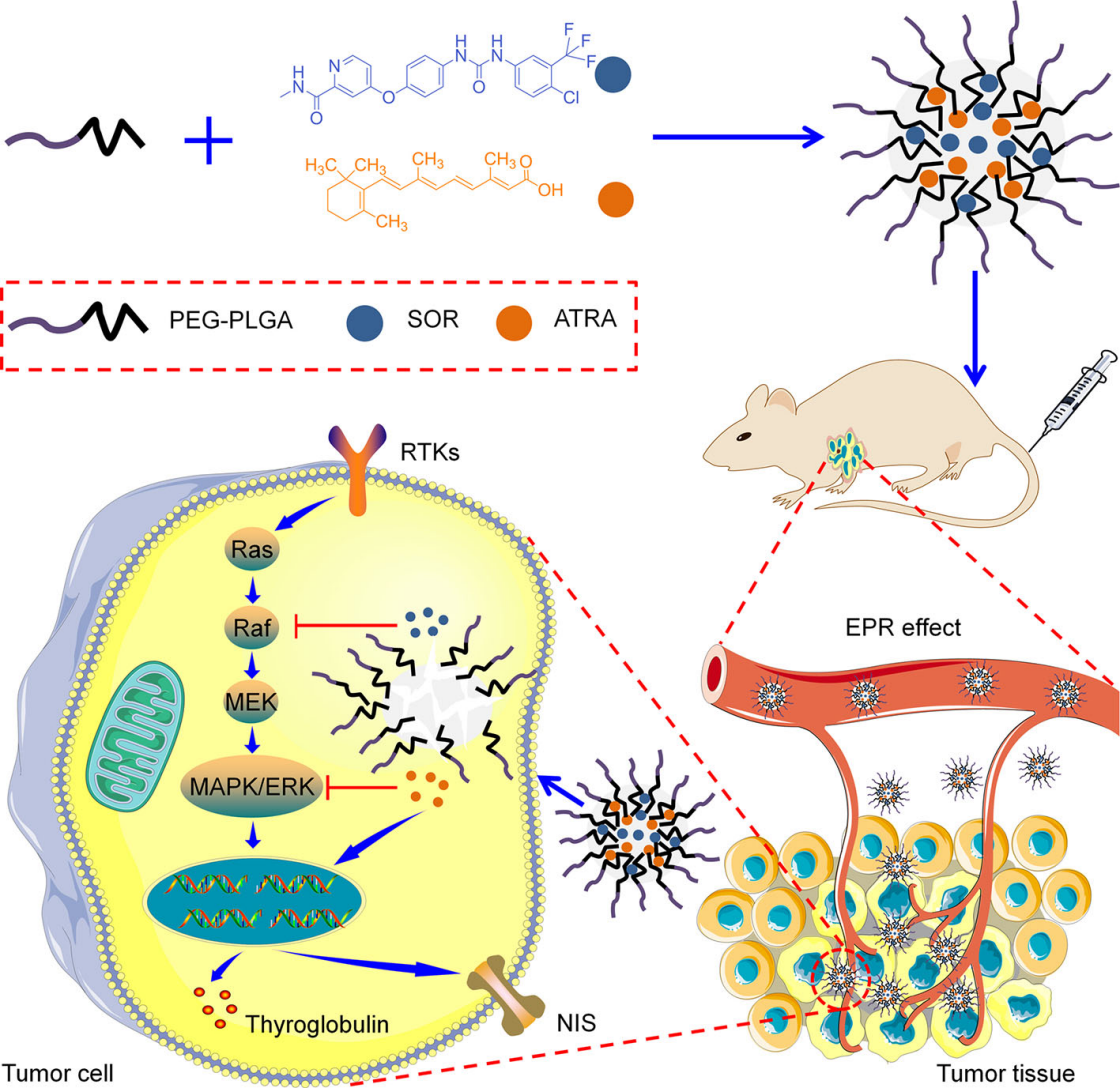
Schematic illustration of the preparation and proposed mechanism of the PEG–PLGA micelles loaded with SOR and ATRA.

**Figure 1:**
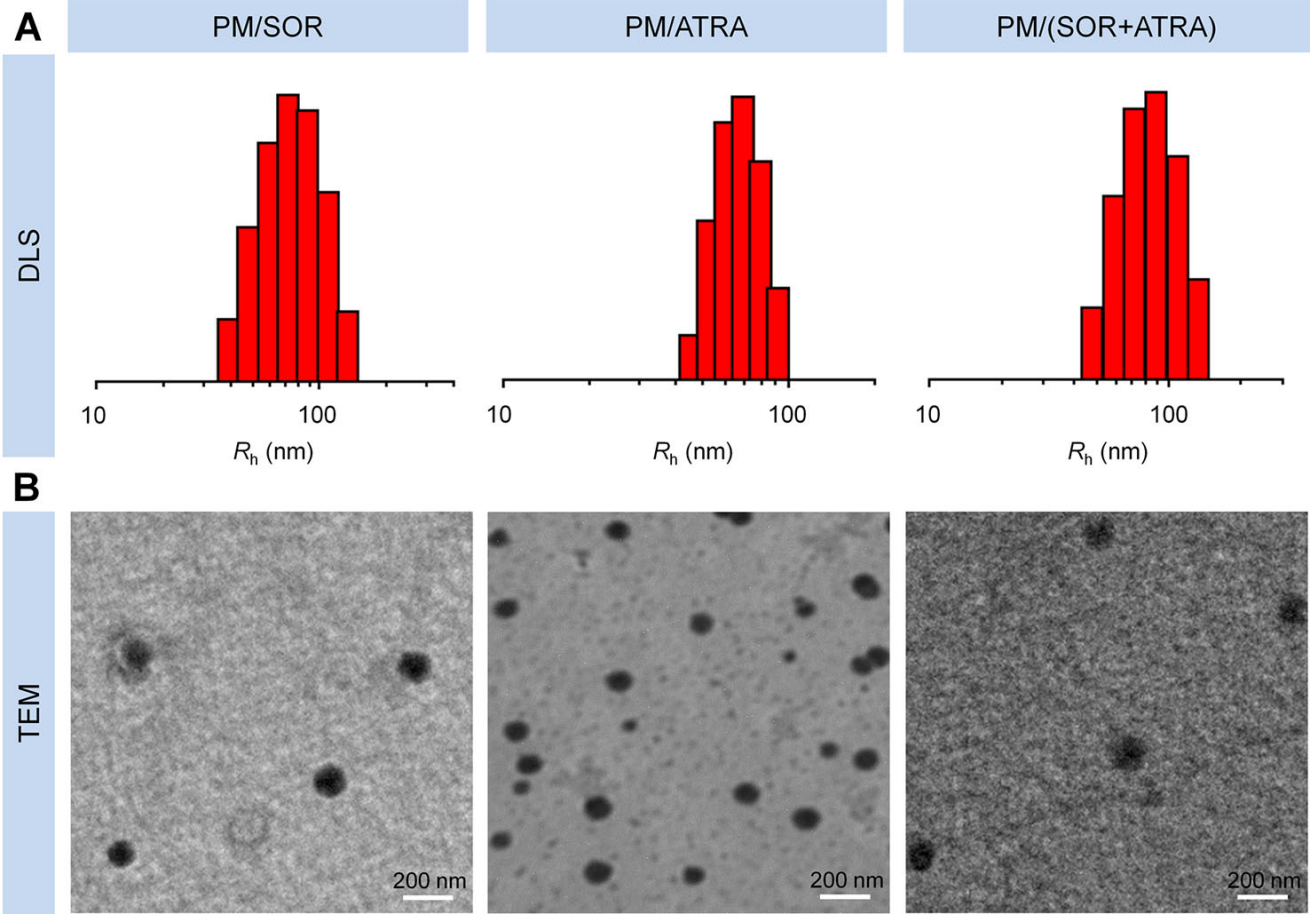
Characterization of drug-loaded PEG–PLGA micelles. **(A)** DLS and **(B)** TEM analyses of PM/SOR, PM/ATRA, and PM/(SOR+ATRA). Scale bar = 200 nm.

PEG–PLGA copolymers were prepared as our previously reported procedure (Li et al., 2013). Briefly, PEG–PLGA copolymers were fabricated through the ring-opening polymerization (ROP) of lactide and glycolide with PEG or tetrahydroxyl-functionalized PEG serving as the initiator and Sn(Oct)_2_ serving as the catalyst. As shown in **Scheme 1**, SOR and ATRA were encapsulated into PEG–PLGA micelles by nanoprecipitation. DLS and TEM characterized the size and morphology of drug loaded micelles. The hydrodynamic radius (*R*_h_) of PM/SOR, PM/ATRA, and PM/(SOR+ATRA) measured by DLS in PBS were about 77.4, 67.1, and 85.1 nm, respectively (**Figure 1A**). Furthermore, TEM showed that all the drug-loaded micelles had uniform spherical morphology and the diameters were about 140.4, 120.8, and 161.4 nm, respectively (**Figure 1B**). All the diameters were less than 200 nm, which was beneficial to their successful accumulation at the tumor site through the enhanced permeability and retention (EPR) effect (Schroeder et al., 2012). Because the size of vascular interendothelial pore is varied, the diameter is a key factor related to the pharmacokinetics and biodistribution of nanoparticles administered intravenously. If the diameters are more than 200 nm, nanoparticles will accumulate in the liver and spleen and be processed by the mononuclear phagocyte system (MPS) cells, which will cause severe loss. However, nanoparticles less than 20 nm in diameter usually have short blood circulation time and high permeation rate, so that they cannot effectively accumulate at the tumor site (Bi et al., 2017).

### *In Vitro* Drug Release and Cell Uptake

**Figure 2:**
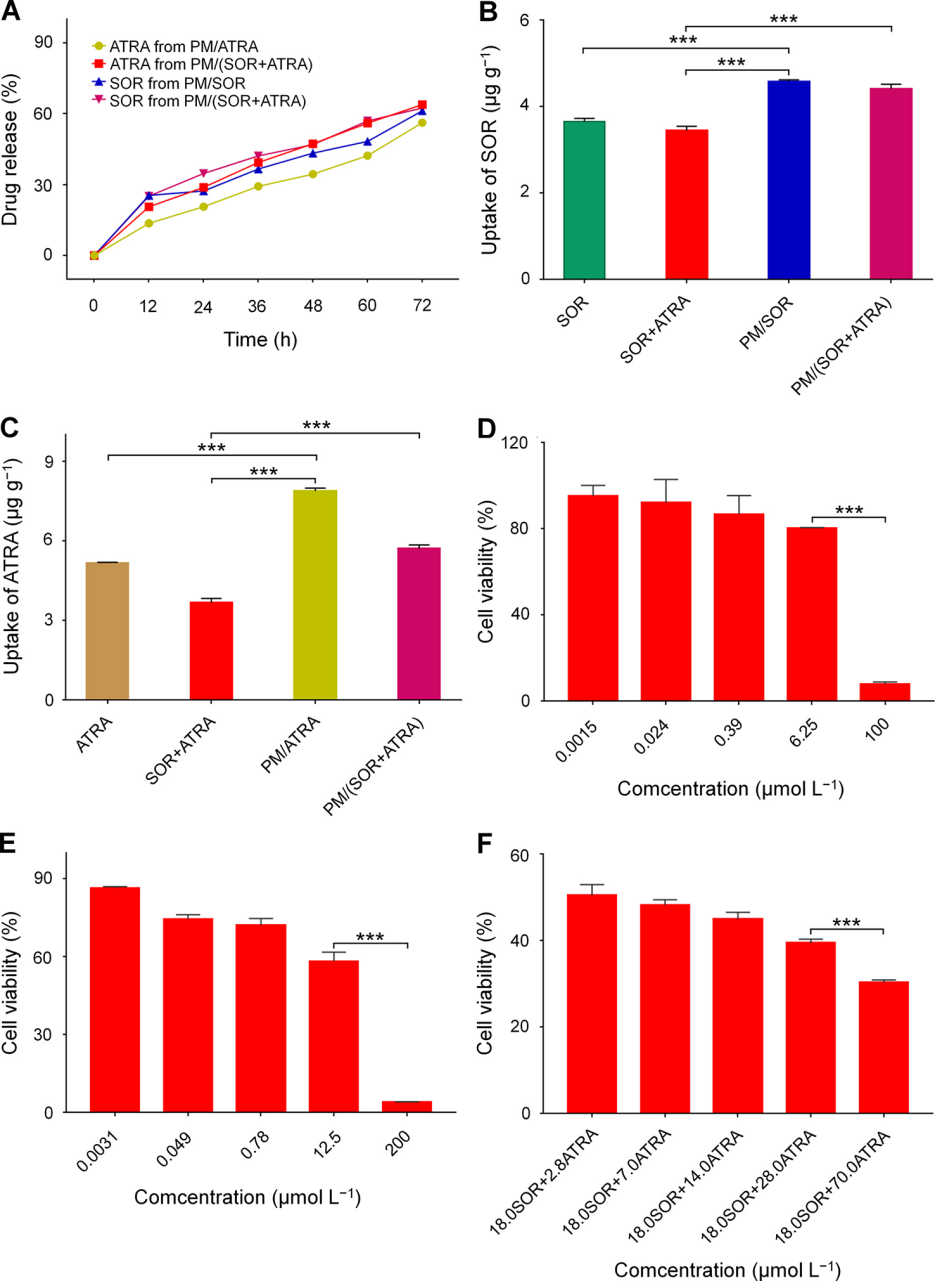
*In vitro* characterization of SOR, ATRA, and drug-loaded PEG–PLGA polymer micelles. **(A)** Release behavior of SOR and ATRA in PBS. Cell uptake of **(B)** SOR and **(C)** ATRA by FTC-133 cells after co-incubation for 2 h. *In vitro* cytotoxicity of **(D)** SOR, **(E)** ATRA, and **(F)** SOR + ATRA on FTC-133 cells. Data are presented as mean ± SD (*n* = 3; ***P < 0.001).

Encapsulation of free drugs in nanocarriers by physical loading or chemical conjugating has been demonstrated to achieve controlled drug release and increase cell uptake effectively (Li et al., 2018). In this study, the *in vitro* drug release behavior of drug-loaded micelles was detected in PBS at pH 7.4 and 37°C. The relationship between UV absorption and drug concentration was determined by standard curve method, which showed good concentration dependence and provided a basis for measuring drug release (**Figure S1**). All the drug-loaded micelles showed slow and sustained drug release, which was shown in **Figure 2A**. In detail, about 61.3% and 62.4% of SOR were released from PM/SOR and PM/(SOR+ATRA) after 72 h of incubation, respectively. Similarly, about 56.3% and 63.9% of ATRA were released from PM/ATRA and PM/(SOR+ATRA) after 72 h of incubation. It was worth noting that a small number of loaded drugs was not released at the end of 72 h, which might be related to the hydrophobic interaction between drugs and micelles, as reported by other studies (Shen et al., 2017). The results verified that the PEG–PLGA micelles could realize the controlled release of two different drugs.

The cell uptake of drug-loaded micelles by FTC-133 cells was evaluated by cytolysis. It could be seen from **Figures 2B, C** that after co-incubation for 2 h, the contents of SOR and ATRA in cells treated with PM/(SOR+ATRA) were about 28.2% and 55.4% higher than those in cells treated with SOR+ATRA, respectively (*P* < 0.001). The contents of SOR and ATRA in cells treated with PM/SOR and PM/ATRA were also significantly higher than those treated with free single or combined drugs (*P* < 0.001). However, there was no obvious difference in intracellular drug content between single drug-loaded micelles and two drugs-loaded micelles. These results indicated that drug-loaded micelles could effectively deliver SOR and ATRA into tumor cells.

### *In Vitro* Cytotoxicity Test

The synergistic antitumor effects of SOR and ATRA on FTC-133 cells were determined by MTT assay. At first, the half-maximal inhibitory concentrations (IC_50_s) of free SOR and ATRA were tested. The cell viability of FTC-133 cells treated with SOR and ATRA was shown in **Figures 2D, E**. The IC_50_s of free SOR and ATRA were 17.9 μmol L^–1^ and 13.9 μmol L^–1^, respectively. To confirm the optimal concentration of SOR and ATRA in synergistic antitumor therapy, we fixed the concentration of SOR at IC_50_ with the concentration of ATRA ranged from 0.2 to 5.0 times of IC_50_. The antitumor effect of SOR combined with ATRA gradually increased as the increase of ATRA concentration, which was shown in **Figure 2F**. The combination of 18.0 μmol L^−1^ SOR and 70.0 μmol L^−1^ ATRA showed the most apparent antitumor effect, and the mass ratio of SOR to ATRA was about 0.4. These results verified the synergistic antitumor effect of SOR and ATRA and provided a reference for the subsequent antitumor animal experiments of drug-loaded micelles. As shown in **Figure S2**, similar antitumor effects were observed in human hepatocellular carcinoma HepG2 cells, suggesting that the combination of SOR and ATRA can also be used for the treatment of other malignant tumors.

### *In Vivo* Antitumor Efficacy Evaluation

**Figure 3:**
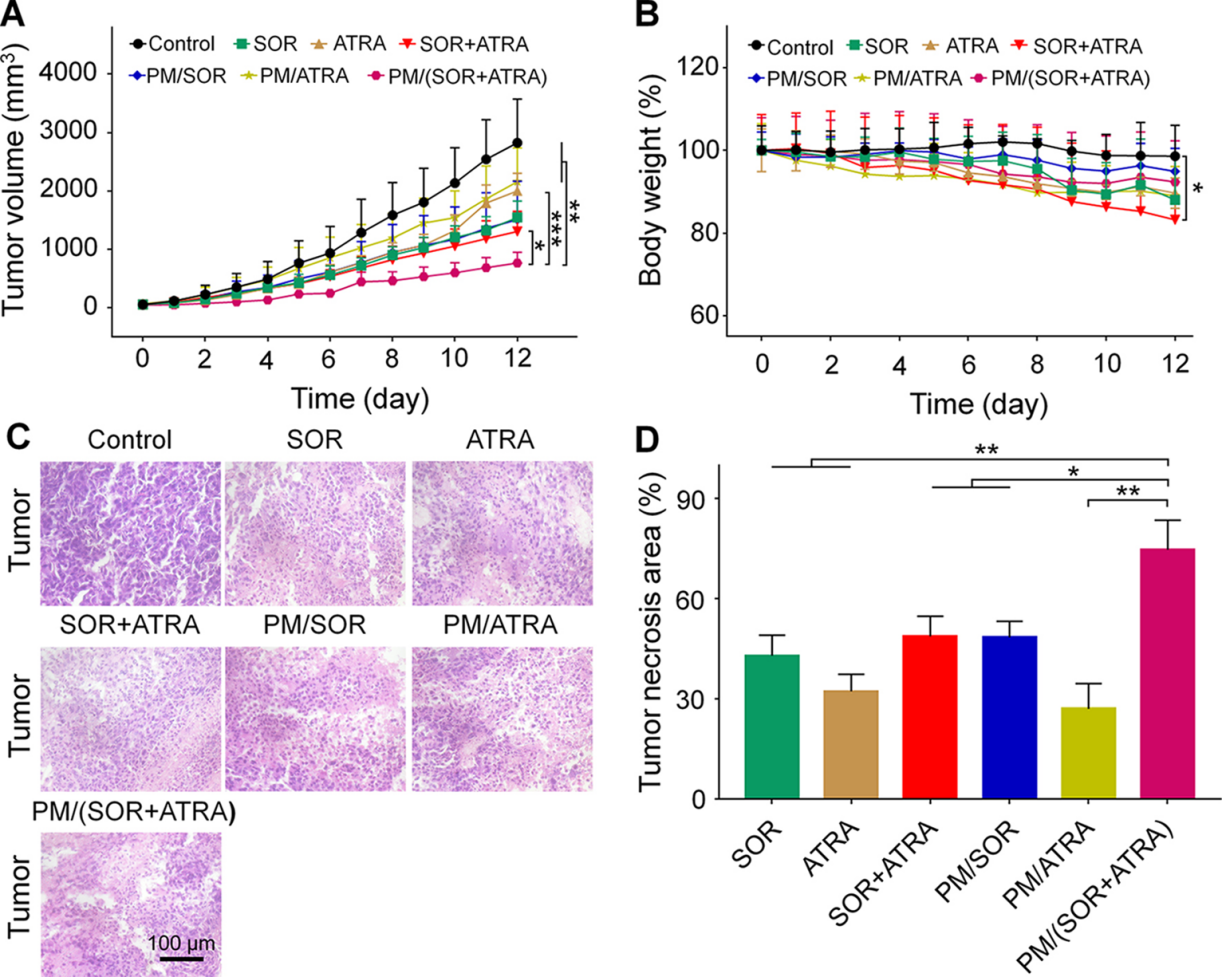
*In vivo* behaviors of different formulations in the FTC-133 thyroid carcinoma-bearing BALB/c mouse model. **(A)** Tumor volumes. **(B)** Body weight of mice treated with different formulations, and the body weight of the mice on day 0 was set to 1. **(C)** H&E staining and **(D)** tumor necrosis area of the tumor tissues obtained from the mouse models treated with different formulations. Data are presented as mean ± SD (in **A**, **B**, *n* = 5, in **C**, **D**, *n* = 3; *P < 0.05, **P < 0.01, ***P < 0.001). Scale bar = 100 μm.

The antitumor efficacy of the drug-loaded micelles was further confirmed in the FTC-133 thyroid cancer-bearing BALB/c nude mice. According to the results of MTT, the combination of SOR and ATRA provided the best tumor inhibition effect when the mass ratio of SOR and ATRA was about 0.4, so we chose the therapeutic dosage of SOR and ATRA to be 10.0 mg (kg BW)^−1^ and 25.0 mg (kg BW)^−1^, respectively. It could be seen from **Figure 3A** that all drug formulations showed different degrees of inhibition on tumor growth, while the tumors in the control group grew rapidly. Notably, the PM/(SOR+ATRA) treatment group exhibited the most significant tumor inhibition effect. The outstanding antitumor effect of PM/(SOR+ATRA) might be attributed to prolonged circulation time, increased aggregation at the tumor site, efficient uptake of tumor cells, and controlled drug release.

The antitumor efficiency of different treatment groups was further evaluated by H&E staining. It could be seen from **Figure 3C** that the tumor cells showed uniform spherical or spindled morphology with a clear nucleus in the control group. In addition, there was almost no apoptotic tissue, suggesting that the proliferation of tumor cells was not affected. In contrast, tumor cells exhibited varying degrees of nucleus pyknotic and cataclastic in the treatment groups, demonstrating that the drugs caused different degrees of apoptosis. The most obvious tumor necrosis was seen in the PM/(SOR+ATRA) treatment group. A quantitative assessment was used to evaluate the necrotic areas. As can be seen from **Figure 3D**, the necrotic areas of SOR, ATRA, SOR+ATRA, PM/SOR, PM/ATRA, and PM/(SOR+ATRA) groups were 43.0, 32.3, 48.8, 48.5, 27.1, and 74.7%, respectively. It was obvious that the tumor necrosis area was the largest in the PM/(SOR+ATRA) treatment group. The results were consistent with the tumor inhibition effect.

### Antitumor Mechanism Study

**Figure 4:**
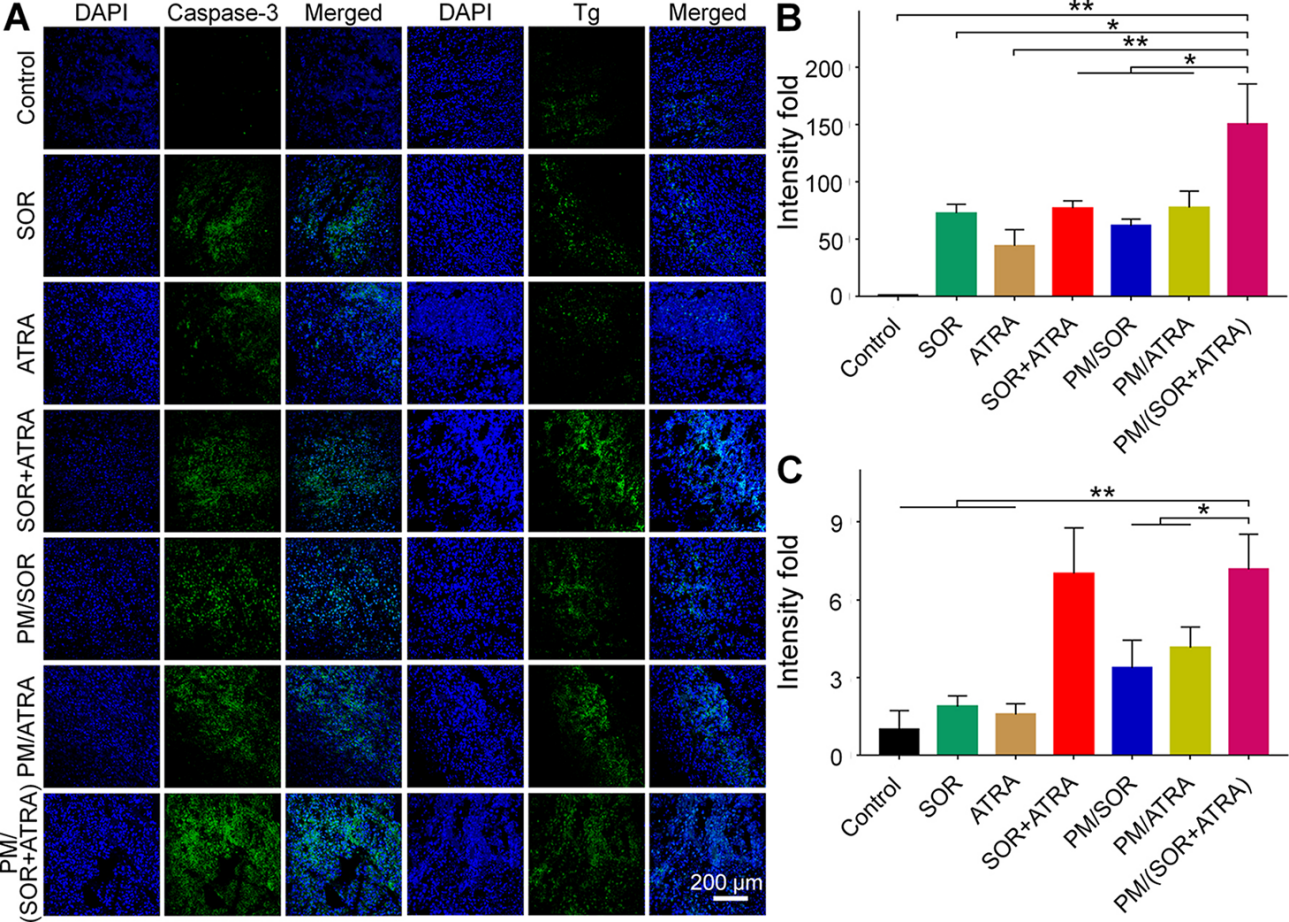
Immunofluorescence staining and semi-quantitative analyses of tumor tissues obtained from the mouse models treated with different formulations. **(A)** Immunofluorescence staining and relative positive areas of **(B)** caspase-3, **(C)** Tg from the semi-quantitative analysis. Data are presented as mean ± SD (*n* = 3; *P < 0.05, **P < 0.01). Scale bar = 200 μm.

**Figure 5:**
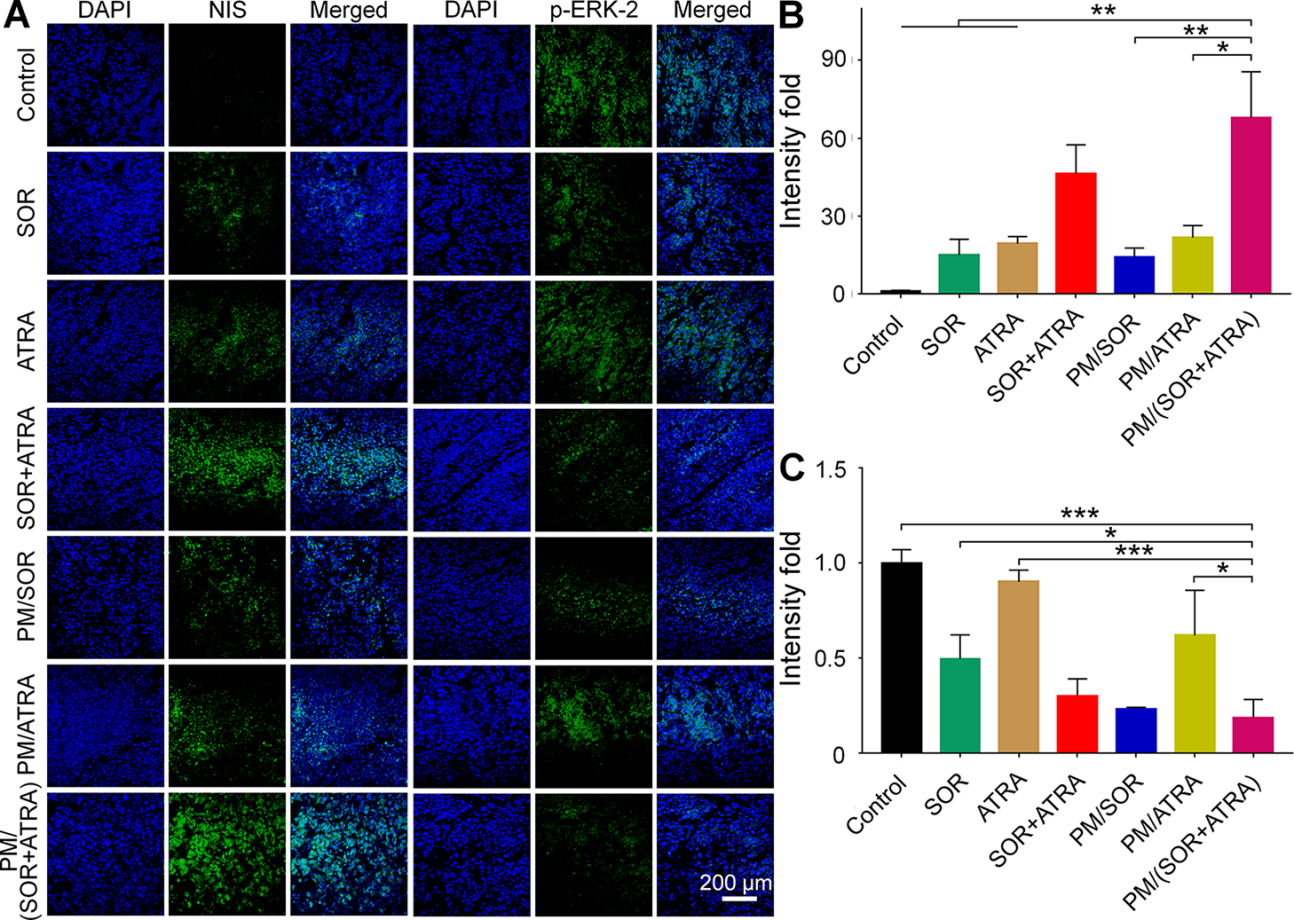
Immunofluorescence staining and semi-quantitative analyses of tumor tissues obtained from the mouse models treated with different formulations. **(A)** Immunofluorescence staining and relative positive areas of **(B)** NIS and **(C)** p-ERK2 from the semi-quantitative analysis. Data are presented as mean ± SD (*n* = 3; *P < 0.05, **P < 0.01, ***P < 0.001). Scale bar = 200 μm.

To validate the possible mechanisms of the combined application of SOR and ATRA, immunofluorescence staining and semi-quantitative analysis were performed. As can be seen from **Figures 4A, B**, the highest level of caspase-3 was expressed in tumor tissues treated with PM/(SOR+ATRA), which verified the advantages of PEG–PLGA micelles delivery and the combined application of SOR and ATRA. DTC cells express several differentiation biomarkers, including Tg and NIS, which are closely related to iodine uptake, thyroid hormone synthesis, and the DTC phenotypes (Bastos et al., 2015). In order to elucidate whether the combination of SOR and ATRA has the potential to induce redifferentiation of thyroid cancer cells, we analyzed the expression levels of Tg and NIS by immunofluorescence staining. Compared with other groups, the PM/(SOR+ATRA) treatment group expressed significantly higher levels of Tg (**Figures 4A, C**) and NIS (**Figures 5A, B**), indicating that SOR combined with ATRA appeared to be a promising differentiation therapy strategy. The expression of Tg and NIS in the group treated with PM/(SOR+ATRA) was 7.2 times and 67.9 times that of the control group, respectively. Mitogen-activated protein kinase (MAPK)/ERK signaling pathway plays an important role in the development of thyroid carcinoma, including cell proliferation and cell survival (Zaballos et al., 2019). The expression of p-ERK2 in the PM/(SOR+ATRA) treatment group was lower than that in other groups (**Figures 5A, C**), indicating that PM/(SOR+ATRA) could effectively inhibit cell proliferation. The above results indicated that PM/(SOR+ATRA) could significantly enhance the apoptosis, promote the differentiation, and inhibit the cell proliferation of FTC-133 thyroid cancer.

### *In Vivo* Security Assessment

**Figure 6:**
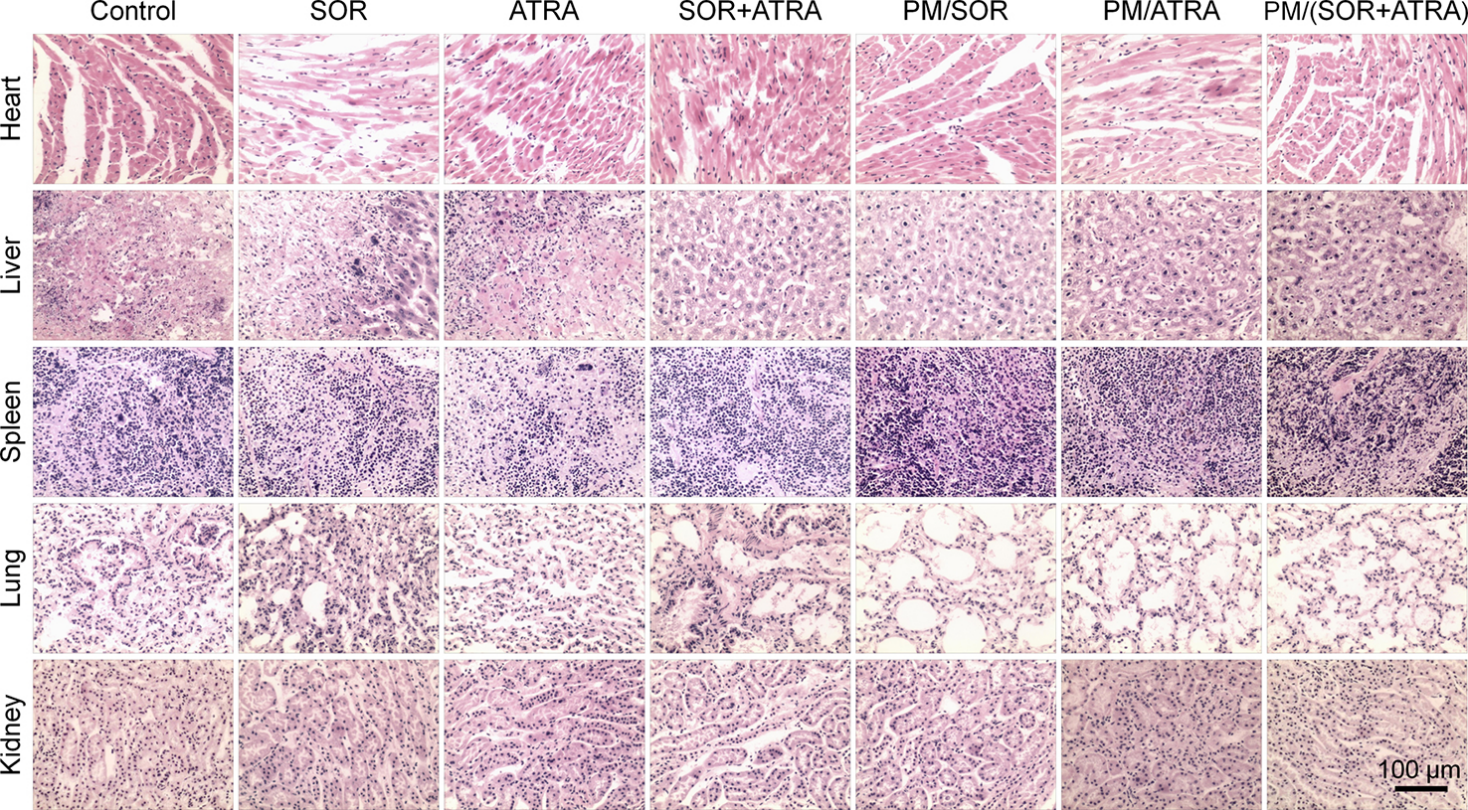
Histological analysis of major organs (*i.e*., heart, liver, spleen, lung, and kidney) obtained from the mouse models treated with different formulations. Scale bar = 100 μm.

Serious side effects often hinder the clinical application of antitumor drugs, so security assessment plays an essential role in clinical practice. The change of body weight is an essential indicator of drug toxicity. **Figure 3B** showed the body weight of mice in different treatment groups. It was worth noting that the free SOR+ATRA treatment group showed the most apparent weight loss (about 16.8%). By contrast, mice in the PM/(SOR+ATRA) treatment group showed only a slight weight loss. In order to further assess the safety of different drug formulations, the main organs (*i.e.*, heart, liver, spleen, lung, and kidney) were collected for H&E analyses. As can be seen from **Figure 6**, various degrees of pulmonary fibrosis, hepatic necrosis, and hypersplenism were observed in the SOR, ATRA, and SOR+ATRA treatment groups. On the contrary, the above pathological changes were slight in the PM/SOR, PM/ATRA, and PM/(SOR+ATRA) treatment groups. All the results further confirmed the satisfactory safety of the drug-loaded micelles.

## Conclusions

The combination of drugs with different mechanisms is a challenging and promising method to solve the limitation of single antitumor drugs. In addition, nanocarriers play a crucial role in enhancing the efficacy of antitumor drugs and reducing their side effects. In this work, we investigated the combination of SOR and ATRA loaded by PEG–PLGA micelles in the treatment of thyroid cancer. The drug-loaded micelles exhibited relatively slow drug release and effective cell uptake. In addition, compared with other treatment groups, the PM/(SOR+ATRA) treatment group had the highest tumor inhibition rate in the FTC-133 thyroid cancer-bearing BALB/c nude mouse model without showing severe systemic toxicity. The expression of NIS and Tg was significantly increased in the groups treated with SOR and ATRA, suggesting that the re-differentiation of thyroid cancer cells may be beneficial for antitumor therapy. All the results verified that the combination of SOR and ATRA and the application of nanocarriers might be an effective treatment for thyroid cancer. Furthermore, the combination of molecular targeted therapy and differentiation therapy may be applied to treat other undifferentiated or poorly differentiated types of malignant tumors, such as liver cancer, colorectal cancer, and gastric cancer.

## Data Availability Statement

All datasets generated for this study are included in the article/**Supplementary Material**.

## Ethics Statement

The animal study was reviewed and approved by the Animal Care and Use Committee at Jilin University.

## Author Contributions

ZL and WX proposed and designed the experiments. SL carried out the experiments with the help of ZL, SD, and YJ. SL and ZL drafted the manuscript and interpreted the data. WX, SD, and YJ revised the manuscript.

## Funding

The work was financially supported by the National Natural Science Foundation of China (Grant Nos. 51603204, 51873207, 51803006, and 51673190) and the Science and Technology Development Program of Jilin Province (Grant Nos. 20190201068JC, 20170101182JC and 20190701004GH). Thanks for the financial support of the Changchun Saikede Medical Device Co, Ltd (Jilin, China).

## Conflict of Interest

The authors declare that the research was conducted in the absence of any commercial or financial relationships that could be construed as a potential conflict of interest.
