# Supplementary material for: Polymer Nanoformulation of Sorafenib and All-Trans Retinoic Acid for Synergistic Inhibition of Thyroid Cancer
Source: Front Pharmacol. 2020 Feb 3;10:1676. doi: 10.3389/fphar.2019.01676 (PMC7008594; doi:10.3389/fphar.2019.01676)
Supplement: Supplementary file 1 [file DataSheet_1.pdf]

Supplementary Material

Polymer Nanoformulation of Sorafenib and All-Trans Retinoic Acid for Synergistic Inhibition of Thyroid Cancer

Shijie Li[1], Shujun Dong[3*], Weiguo Xu[4*], Yang Jiang[2], Zhongmin Li[2,4*]

[1] Department of Thyroid Surgery, China-Japan Union Hospital of Jilin University, 126 Xiantai Street, Changchun 130033, P. R. China

[2] Department of Gastrointestinal Colorectal and Anal Surgery, China-Japan Union Hospital of Jilin University, 126 Xiantai Street, Changchun 130033, P. R. China

[3] VIP Integrated Department, School and Hospital of Stomatology, Jilin University, 1500 Qinghua Road, Changchun 130021, P. R. China

[4] Key Laboratory of Polymer Ecomaterials, Changchun Institute of Applied Chemistry, Chinese Academy of Sciences, 5625 Renmin Street, Changchun 130022, P. R. China

*Correspondance:

Zhongmin Li

lizhongmin1211@126.com

Weiguo Xu

wgxu@ciac.ac.cn

Shujun Dong

dsj@jlu.edu.cn

Supplementary Figures

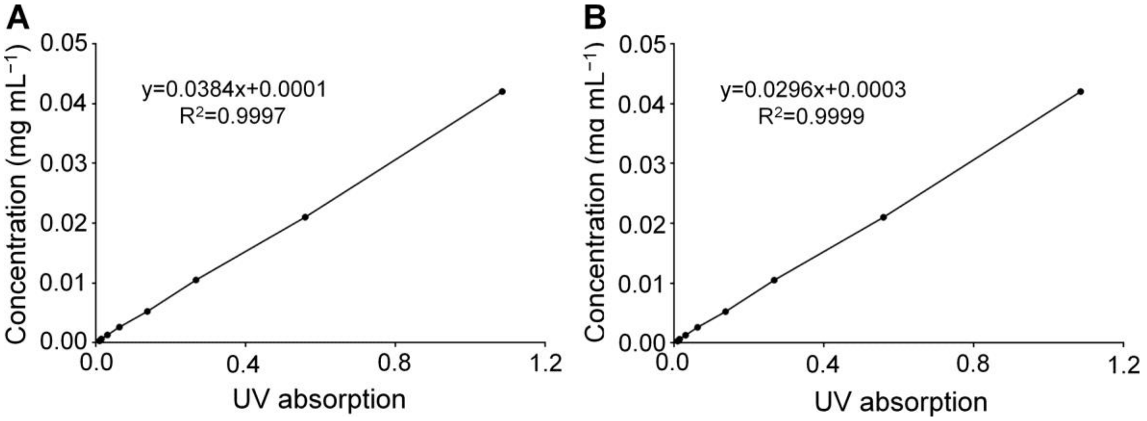

Supplemental Figure 1. The standard curves of UV absorption with concentrations of SOR (A) and ATRA (B).

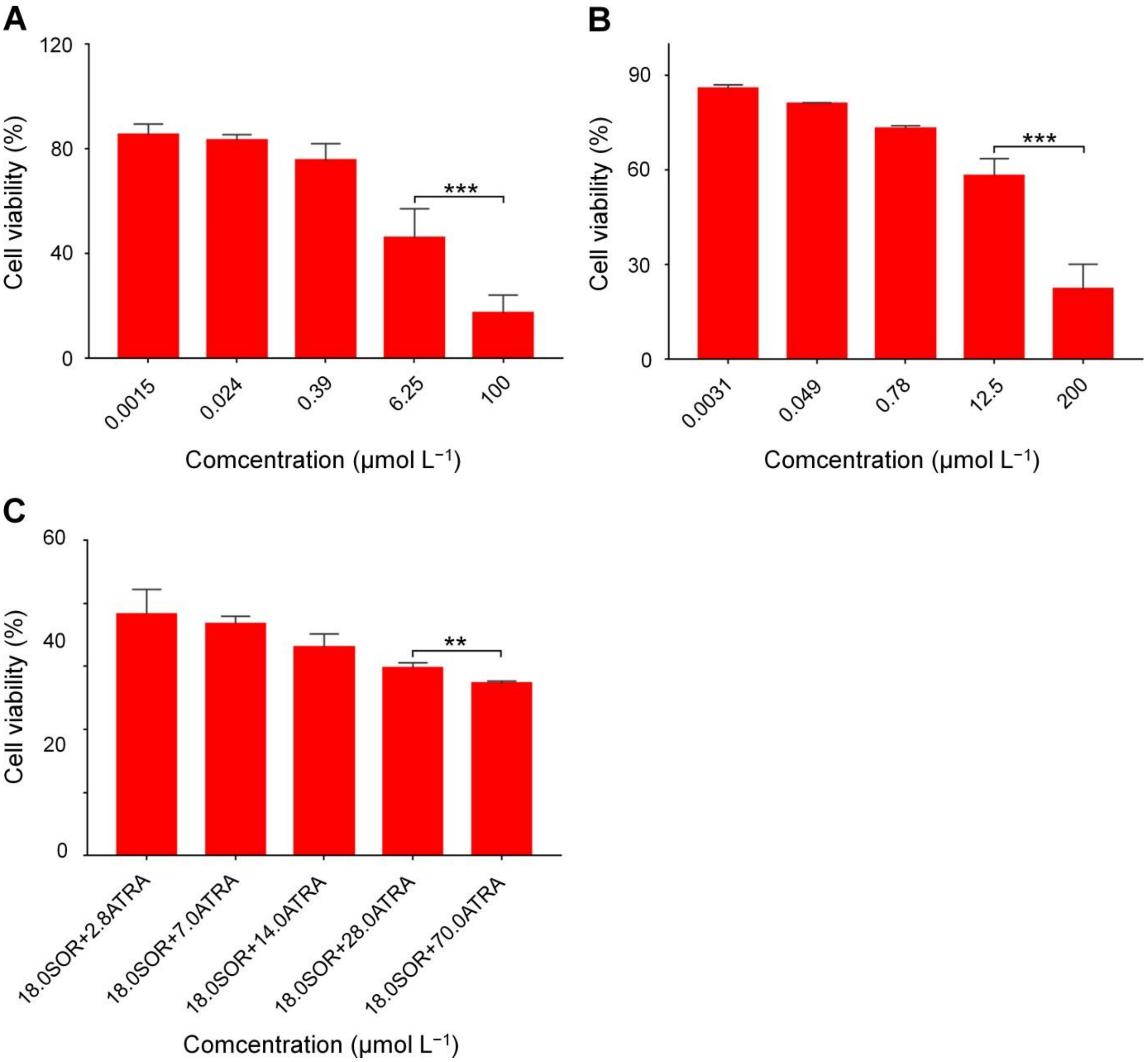

Supplemental Figure 2. Evaluation of antitumor effect of SOR and ATRA on HepG2

cells *in vitro*. *In vitro* cytotoxicity of (A) SOR, (B) ATRA, and (C) SOR + ATRA. Data are presented as mean ± SD (n = 3; *P < 0.05, **P < 0.01, ***P < 0.001).
